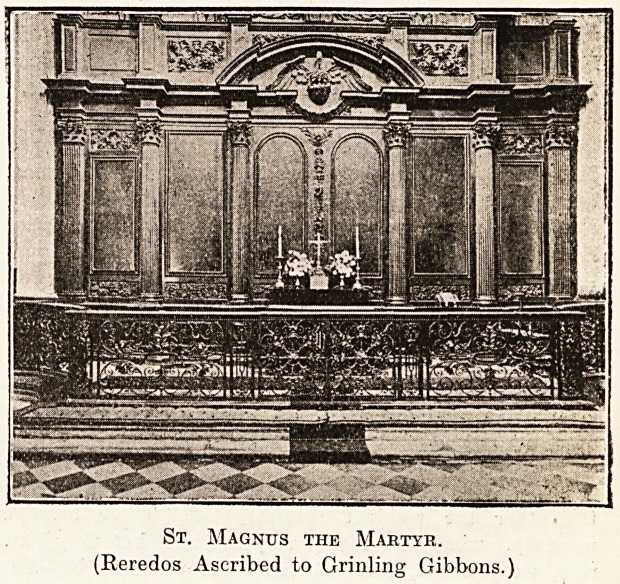# St. Magnus the Martyr and Hospital Sunday

**Published:** 1915-06-12

**Authors:** 


### ST. MAGNUS THE MARTYR AND HOSPITAL SUNDAY.

The church of St. Magnus the Martyr, where the Lord Mayor attended a special service on Thursday, June 10, on behalf of the Metropolitan Hospital Sunday Fund, claims the proud position of collecting the largest amount of any City church through its congregational collections for Hospital Sunday. It is one of the City churches built by Sir Christopher Wren after the Great Fire of 1666. Close by the spot where the Monument "lifts its tall head," and at the bottom of Fish Street Hill, the church stood on the approach to London Bridge, and the porch and tower projected into the street. They were ingeniously contrived, however, by Wren, so that by piercing the lower part of the tower the footway to the bridge actually passed through the porch. But when, in 1825, the new bridge was built it was placed some 200 feet further to the west, and part of the old roadway was enclosed and made into a churchyard.

The identity of St. Magnus is not indisputably established. By some he is supposed to have been a Danish king, and it is interesting in this connection to recall the great battle on this spot between Ethelred the Unready and Sweyn, King of Denmark, when many of the Danes were drowned, as the chronicler tells us. By others, however, St. Magnus is supposed to have been an early Christian, who suffered martyrdom at Cæsarea under the Emperor Aurelian in the third century. The old church, of which no trace now remains, was, from its position on the great highway to London, of considerable importance, and contained the tombs of some well-known people. Among these was Henry Yeveley's, the great master-mason of the end of the fourteenth century. It is to Yeveley that we owe the design of the nave of Westminster Abbey and the very unusual feature that his work carried on and continued the plan and design of the choir, which had been built over a hundred years previously. Yeveley also designed and directed the mason's work for the reconstruction of Westminster Hall. But the greatest name connected with St. Magnus is that of Miles Coverdale (d. 1568), who, as his epitaph records, "convinced that the pure Word of God ought to be the sole rule of our faith and guide of our practice . . . with the view of affording the means of reading and hearing in their own tongue the works of God, not only to his own countrymen, but to the nations that sit in darkness and to every creature wheresoever the English language might be spoken," prepared and printed the first English-printed version of the Bible in 1535. It is from this version that we derive the Prayer Book Psalter. Coverdale was Rector of St. Magnus for a few years, and was first buried in St. Bartholomew's Exchange, but when that church was pulled down in 1840 his remains were brought to St. Magnus and re-interred near the altar.

In 1666 came the Great Fire, which began, as Mrs. Markham taught us, in Pudding Lane and ended at Pie Corner. But, however that may be, Pepys records how, going down "with my heart full of trouble to the Lieutenant of the Tower, he tells me that it began this morning in the King's baker's house in Pudding Lane, and that it hath burned down St. Magnus Church and most part of Fish Street already. So I down to the water-side and there got a boat, and through bridge, and there saw a lamentable fire." And how a little later he met "my Lord Mayor like a man spent, with a handkercher about his neck, who cried, like a fainting woman, 'Lord! What can I do? I am spent: people will not obey me. I have been pulling down houses; but the fire overtakes us faster than we can do it.'" A curious relic of the fire remains in the old manual fire engines and leather buckets, now preserved in the church, which probably date from the seventeenth century. Nothing, however, remained of the church, and in 1676 Sir Christopher Wren, perhaps remembering that one of his family had been a former rector, erected the present church on the site, and the steeple, which is one of the most beautiful of his works, was added in 1705. The interior was described at the time as "suitable for a place of Divine worship, being elegant without gaudiness, and imposing without finery." But the finely proportioned altar-piece, with its carving by Grinling Gibbons and its Sussex wrought-iron Communion rails, the carved pulpit and organ case, are beautiful examples of seventeenth and early eighteenth century work. The organ, which was presented in 1712 by Alderman Sir Charles Duncombe, was built by Abraham Jordan, and, as he informed the public at the time, "is adapted to the art of emitting sounds by swelling the notes, which never was in any organ before." The outside clock was also the gift of the Alderman. One or two tablets may, perhaps, be mentioned—one to Mrs. Amelia Corderoy (d. 1789), who, unable to survive her husband, "died a victim of conjugal affection"; and a tombstone in the churchyard to Robert Preston (d. 1730), "late Drawer at the Boar's Head Tavern in Great Eastcheap," in this parish, with the following quaint inscription:—

St. Magnus the Martyr.
(Reredos Ascribed to Grinling Gibbons.)

"Bacchus, to give the Toping World surprize,
Produc'd one Sober Son, and here he lies;
Tho' nurs'd among full Hogsheads, he defyd
The charms of Wine and ev'ry vice beside.
O Reader, if to Justice thou'rt inclin'd,
Keep honest Preston daily in thy mind.
He drew good Wine, took care to fill his pots,
Had sundry virtues that outweigh'd his faults.
You that on Bacchus have the like dependance
Pray copy Bob in Measure and Attendance."

But the Boar's Head recalls other memories; for was it not there that Falstaff made merry with Prince Hal, and told the story of the great encounter with the stout rogues in buckram?

## Figures and Tables

**Figure f1:**